# SP-WVD with Adaptive-Filter-Bank-Supported RF Sensor for Low RCS Targets’ Nonlinear Micro-Doppler Signature/Pattern Imaging System

**DOI:** 10.3390/s22031186

**Published:** 2022-02-04

**Authors:** Harish C. Kumawat, A Arockia Bazil Raj

**Affiliations:** Electronics Engineering, Defence Institute of Advanced Technology, Pune 411025, India; harish_pee18@diat.ac.in

**Keywords:** CW RF sensor, micro-Doppler profile imaging, short-time Fourier transform, wavelet transform, smoothed pseudo-Wigner–Ville distribution with adaptive decomposition filter

## Abstract

In this study, the authors present the accurate imaging of the behavior of simultaneous operations of multiple low radar cross-section (RCS) aerial targets. Currently, the popularity of low RCS targets is increasing day by day, and detection and identification of these targets have become critical issues. Micro-Doppler signatures are key components for detecting and identifying these low RCS targets. For this, an innovative approach is proposed along with the smooth pseudo-Wigner–Ville distribution (SP-WVD) and adaptive filter bank to improve the attenuation of cross-term interferences to generate more accurate images for the micro-Doppler signatures/patterns of simultaneous multiple targets. A C-band (5.3 GHz) radio-frequency (RF) sensor is designed and used to acquire the micro-Doppler signatures of aerial rotational, flapping, and motional low RCS targets. Digital pipelined-parallel architecture is designed inside the Xilinx field-programable gate array (FPGA) for fast sensor data collection, data preprocessing, and interface to the computer (imaging algorithm). The experimental results of the proposed approach are validated with the results of the classical short-term Fourier transform (STFT), continuous wavelet transform (CWT), and smooth pseudo-Wigner Ville distribution (SP-WVD). Realistic open-field outdoor experiments are conducted covering different simultaneous postures of (i) two-/three-blade propeller/roto systems, (ii) flapping bionic bird, and (iii) kinetic warhead targets. The associated experimental results and findings are reported and analyzed in this paper. The limitations and possible future research studies are also discussed in the conclusion.

## 1. Introduction

A radio-frequency (RF) sensor uses electromagnetic waves for the detection of objects (known as targets). In general, there are basically three types of RF sensors: continuous-wave (CW), pulsed-wave, and frequency-modulated CW (FMCW) sensors [1]. CW and FMCW sensors require low power (when compared to pulsed radar sensors) for the given range due to their continuous modulated or unmodulated transmission; hence, they can be built with relatively simple circuits [2,3]. Recent technology developers are building low radar cross-section (RCS) targets such as drones, unmanned aerial vehicles (UAVs), quadcopters, bionic birds, mini-helicopters, mini-bombs, and mini-kinetic warhead structures [4,5,6,7]. Due to low RCS and other electronic sophistication, these kinds of targets are coming up for their extensive use in various defense and civil applications such as surveillance, spying, air traffic control, law and order maintenance, delivery of materials/goods, aerial mini-missile/mini-bomb/mini-warhead guidance, and aerial network formation for military target tracking/data-exchange applications [5,8,9,10]. The signatures of propeller blades’/rotors’ rotations, wing flapping, and/or kinetic warhead motions are the only key components to detect these types of targets [11,12,13]. To detect these low RCS targets, sensing and processing the micro-Doppler signature corresponding to the different behaviors of these targets and imaging their activities have become significant. Different time-frequency analysis techniques are used to detect the micro-Doppler of the targets [8,14,15].

Several studies are carried out currently either on improving the sensitivities of the RF receiver chain [16] or on increasing the computation accuracy of signal processing/imaging algorithms [17,18] to pick up and process the micro-Doppler signature/pattern profiles generated by these low RCS targets [19,20].

A few recent works, more relevant to our research work, found in the literature are as follows. S. Rahman and D. A. Robertson experimentally studied the micro-Doppler signature of a drone and a bionic bird. These targets were operated in the front of two radars (24 GHz and 94 GHz) within a 30 m range. The experiment was limited only to the generation of a micro-Doppler signature for direct rotational and flapping motions. The accuracy of spectrogram images generated by both radars was compared. Any combined (multifrequency) micro-Doppler effects were not analyzed [5]. V. C. Chen presented various models for the micro-Doppler effects in radar. In his book, he designed theoretical/geometrical models for different kinds of targets: rigid (pendulum, rotors blades, and wind turbine) targets and non-rigid (human, wing flapping, and quadrupedal animal motion) targets. In our experiment, we used these models, and their results exhibited a high degree of disagreement with the measurement results. This is because the models were designed considering only the main geometrical parameters and ideal conditions/values for both sensor and target. Furthermore, this book only addresses the theoretical micro-Doppler effects of individual targets and not the combined (multifrequency) micro-Doppler effects [8].

J. Gong et al. conducted an indoor experiment inside an anechoic chamber with a bionic bird to distinguish its flapping and gliding motions. The target was treated as a corner reflector, a modulated signature was taken as flapping, and no signal was taken as a gliding signature [12]. M. Jian et al. used a commercially available 24 GHz radar module and used it for the detection of the rotational/radial velocity of single and four rotors in a closed environment. In their work, the targets were individually operated at high radial velocity and extracted both radial and angular components using the results of the short-time Fourier transform (STFT) algorithm [13]. B. Y. Su et al. used wavelet transform (WT) to detect human falls within a room using a ceiling-mounted Doppler radar operated at 5.8 GHz (C-band). The time-frequency image was used to classify human postures including only standing or falling down [21].

P. Meena et al. used the variational mode decomposition (VMD) technique to suppress the cross-term interference generated in the Wigner–Ville distribution (WVD). The proposed technique was validated with simulated non-stationary multifrequency signals [22]. N. Whitelonis et al. presented a compressed sensing joint time-frequency (CSJTF) technique to analyze radar signals. The method was tested against the simulated results, and the obtained resolution was the same as the STFT results [23]. Wang et al. proposed a variational mode decomposition (VMD)-based WVD approach for cross-term interference suppression. They decomposed a multifrequency seismic signal using VMD and WVD algorithms. Everything was performed on computer simulations; no experimental test was conducted [24].

P. Klaer et al. have presented drone detection and single-propeller and multi-propeller drone identification based on micro-Doppler signature using rotary drone HERM line spectrum. For micro-Doppler signal collection, they have used C-band CW radar and W-band FMCW radar. For time-frequency analysis, they have used only STFT [25]. Z. Xiao and Z. Yan have presented time-frequency analysis and CNN-based radar emitter identification method. They have used STFT and k-means algorithm for TF spectrum calculation and CNN for identification. They have considered STFT, WVD, smooth pseudo-Wigner Ville Distribution (SP-WVD), Choi–Williams distribution (CWD), and bispectrum-based methods for time-frequency response comparison. The proposed technique was validated with simulated results [26]. Y. Zhou et al. have proposed large micro-motion target detection and parameter estimation technique for synthetic aperture radar (SAR) using Hough transform. In the proposed method, to obtain a time-frequency response of the azimuth radar echo signal, they used STFT. After that, the Hough transform is used for target detection and parameter estimation. Large fixed-angle reflectors are used as targets for the experiments [27]. Á.D.de Quevedo et al. have presented Drone detection and radar-cross-section measurements using FMCW radar named RAD-DAR [28].

In one of our recent publications, we reported the effects of micro-Doppler signatures generated by a walking/jogging/cycling person. New techniques were proposed for the computation of revolutions per minute (RPM) and oscillations per minute (OPM) using the micro-Doppler frequency produced by rotor blades and oscillating bodies (e.g., pendulum), respectively. The proposed techniques were experimentally validated against standard measurement results [29].

To the best of our knowledge, in the literature, micro-Doppler signatures of rotational/flapping targets are obtained/analyzed separately (not the combined effects) using different types of RF sensor systems and signal processing techniques. In most of the literature, the targets are simulated or operated within an anechoic chamber or a small room, which shows less agreement with the results of outdoor experiments. Therefore, building an RF sensor with an accurate micro-Doppler signature/pattern imaging technique (precise multifrequency signal decomposition) and using it to sense/recognize the combined (simultaneous activities of more than one aerial target) behaviors of real open-field outdoor-operated rotational/flapping/kinetic warhead targets became significant. For accurate micro-Doppler signature/pattern imaging, we have used SP-WVD with an adaptive filter bank to attenuate cross-term interferences, which is produced by the simultaneous operation of multiple targets, which is the main contribution presented in this paper.

**Figure 1:**
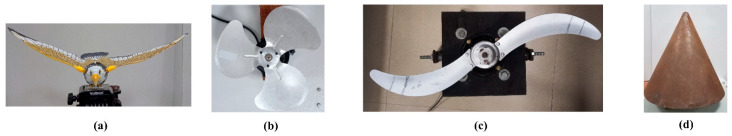
Low Radar cross−section (RCS) (**a**) flapping, (**b**,**c**) rotational, and (**d**) kinetic warhead motion targets used in work reported in this paper.

For this purpose, a CW RF sensor system was developed, and four different targets were operated in the real open-field environment at various ranges in front of the sensor: a bionic bird, three-/two-blade rotational drone propeller systems, and kinetic warhead structure targets, as shown in Figure 1. The sensor echo signals were applied to the micro-Doppler signature/pattern imaging [30,31] algorithms. The main specifications of these low RCS targets used in our work are given in Table 1. The bird flapping speed, the RPM of rotors, and the warhead motion linear speed were controlled remotely by controlling the voltage applied across them [32,33,34]. Load voltage controls were performed using a remote-control module with a potentiometer (pot.) and silicon control rectifier (SCR) circuits/knobs [32,35]. The real-time-acquired sensor signals were applied to two different algorithms: the STFT [36] and continuous wavelet transform (CWT) [37]. The accuracy of the micro-Doppler signature/pattern images designed using the results of these algorithms did not gain appreciation due to their inherent limitations listed in [38,39,40].

Further proceeding with the manual or automated behavior detection/recognition of these low RCS targets using the results of these two algorithms becomes difficult. Therefore, we preferred the SP-WVD technique [22] applied on the sensor signal, and a reasonable improvement was attained in imaging accuracy. However, this SP-WVD technique introduced cross-term interference due to the computations involved in the algorithm [24,40,41]. To attenuate this cross-term, an innovative approach was proposed, combining the adaptive decomposition filter bank with the SP-WVD, and the results yielded appreciable attenuation of the cross-term interference. Therefore, the adaptive-decomposition-filter-bank-applied SP-WVD algorithm was used throughout our work, and the experimental results obtained for the RF sensor performance validation and the targets’ behavioral imaging using the proposed innovative approach are presented.

The rest of the paper is organized as follows: Section 2 explains the mathematical description of the algorithms (STFT, CWT, SP-WVD, and improved SP-WVD) applied in this work, Section 3 briefly describes the design of the RF sensor and sensor data processing/imaging techniques, Section 4 presents experimental results and data analysis, Section 5 provides a state-of-the-art (SOTA) comparative discussion, and Section 6 provides the conclusion.

## 2. Mathematical Descriptions

The necessary concepts and relevant mathematics related to the algorithms (STFT, CWT, and SP-WVD) considered in this work for comparative analysis are given in this section. More details on these algorithms can be found in [21,36,41,42].

### 2.1. Short-Term Fourier Transform

The power spectrum for the entire frequency of a sensor signal is generated using fast Fourier transform (FFT), which is the fastest algorithm for computing the discrete Fourier transform (DFT) [43]. The DFT of an *N*-point sequence for the *k* frequency levels is computed as follows:

(1) $$X\left[k\right]=\overset{N-1}{\underset{n=0}{\sum }}x\left[n\right]e^{-j\left(2\pi nk/N\right)}$$

where *k* = 0, 1…., *N* − 1, *x*[*n*] denotes the *n*th sample of the sensor signal, and *N* represents the length of the sequence. The STFT can be derived from the FFT to perform the time-frequency localization of non-stationary signals. The STFTs for continuous and discrete-time signals are as follows [36]:

(2) $$X(\tau ,\;\omega )=\int_{-\infty }^{+\infty }x\left(t\right)w\left(t-\tau \right)e^{-j\omega t}dt\;\mathrm{and}$$

(3) $$X\left[n,k\right]=\overset{\infty }{\underset{m=-\infty }{\sum }}x\left[m\right]w\left[n-m\right]e^{-j2\pi mk/N}$$

where *w*(*t*) and *w*[*n*] represent the window function for analog and discrete-time signals, respectively. The STFT uses a fixed window (thus fixed resolution) to compute the time-frequency response of the signal. A narrow window provides good time resolution and poor frequency resolution, while a wide window provides poor time resolution but good frequency resolution [38]. Thus, one should choose a trade-off between time and frequency resolution in the STFT. The squared magnitude of the STFT provides the spectrogram (S) [36,38] as follows.

(4) $$S={\left|X\left(\tau ,\;\omega \right)\right|}^{2}={\left|X\left[n,k\right]\right|}^{2}$$

The time-and-frequency trade-off STFT results form a matrix of A columns and B rows, where A represents the time resolution and B denotes the frequency resolution [32,36]. The values of A and B are obtained by the following:

(5) $$A=⎣\frac{\left(N-\;C\right)}{\left(D\;-\;C\right)}⎦\;\mathrm{for}\;\mathrm{either}\;\mathrm{window}\;\mathrm{is}\;a\;\mathrm{scalar}\;\mathrm{or}\;a\;\mathrm{vector}$$

(6) $$B=\{\begin{array}{c} \left(\frac{\mathrm{nfft}}{2}+1\right)\;\;\;\;\;\;\;\mathrm{if}\;\mathrm{nfft}\;\mathrm{is}\;\mathrm{even}\\ \left(\frac{\mathrm{nfft}+1}{2}\right)\;\;\;\;\;\;\;\;\;\;\mathrm{if}\;\mathrm{nfft}\;\mathrm{is}\;\mathrm{odd} \end{array}$$

where C represents the length of overlapped samples, D represents the length of the window, and nfft represents the number of DFT points. Therefore, STFT has fixed-time and fixed-frequency resolution problems. The STFT window length is 512 samples (51.2 ms) in our work.

### 2.2. Wavelet Transform

CWT is another approach for time-frequency analysis; in fact, it overcomes the time-frequency resolution issue of the STFT to some extent [37]. The continuous wavelet transform is defined as follows [37]:

(7) $$WT_{x}^{\Psi }\left(\tau ,s\right)=\int_{-\infty }^{+\infty }x\left(t\right)\Psi_{\tau ,s}{}^{*}\left(t\right)dt$$

where $\Psi_{\tau ,s}\left(t\right)=\frac{1}{\sqrt{s}}\Psi \left(\frac{t-\tau }{s}\right)$.

Here, *Ψ*(*t*) is the transforming function called the mother wavelet, and $\Psi_{\tau ,s}\left(t\right)$ is the dilated/translated mother wavelet. *τ* and *s* are the translation and scale parameters, respectively. The movement of the wavelet along the time axis is governed by translation parameter *τ*, and the dilation (*s* > 1)/contraction (*s* < 1) of the wavelet is governed by the scale parameters. The analytic wavelet is used in this work with CWT, the complex-valued wavelet for which its Fourier transform vanishes for negative frequencies. The Morlet wavelet, given in Equation (8), is used as the mother wavelet with the central frequency *f_c_* as follows [40].

(8) $$\Psi \left(t\right)\;=\frac{1}{\pi^{1/4}}e^{i2\pi f_{c}t}e^{-t^{2}/2}$$

### 2.3. Smoothed Pseudo-Wigner–Ville Distribution with an Adaptive Filter Bank

WVD provides a high-resolution time-frequency representation of a multifrequency non-stationary signal [24]. WVD is a quadratic distribution that provides simultaneous time and frequency localization. This distribution is also effectively used in signal visualization, detection, and estimation [40]. For a continuous signal *x*(*t*) and a discrete signal *x*[*n*], the WVD is given by the following [22,41].

(9) $${\mathrm{WVD}}_{x}(t,\;\omega )=\frac{1}{2\pi }\int_{-\infty }^{+\infty }x\left(t+\frac{1}{2}\tau \right)x^{*}\left(t-\frac{1}{2}\tau \right)e^{-j\omega \tau }d\tau \;\;\mathrm{and}$$

(10) $${\mathrm{WVD}}_{x}(n,\;k)=\sum_{m=-N}^{N}x\left(n+\frac{m}{2}\right)x^{*}\left(n-\frac{m}{2}\right)e^{\frac{-j2\pi km}{N}}$$

Even though WVD provides a high-resolution time-frequency representation, the cross-term interference (that appears more for a multifrequency signal) limits its accuracy. For example, let *x*(*t*) be the sum of two signals $x_{1}\left(t\right)$ and $x_{2}\left(t\right)$; then, the WVD for this signal is given by the following [42,43]:

(11) WVD (t, ω)=WVD11(t, ω)+WVD22(t, ω)+2Re{WVD12(t, ω)}

where Re represents the real part. In Equation (11), the first two terms represent the actual signal, and the last term represents their cross-term interference. The cross-term interference badly influences the results of the WVD algorithm in identifying original frequencies present in a multifrequency signal [41]. Therefore, attenuating cross-term interference becomes significant, which in this work is performed by incorporating the smoothed pseudo-operation in the results of the WVD. The SP-WVD is given as follows:

(12) $$SPWVD_{x}^{G,H}\left(t,f\right)=\int_{-\infty }^{+\infty }H\left(\tau \right)\int_{-\infty }^{+\infty }G\left(s-t\right)x\left(s+\frac{1}{2}\tau \right)x^{*}\left(s-\frac{1}{2}\tau \right)e^{-j2\pi f\tau }dsd\tau$$

where *H* and *G* are the frequency-smoothing and time-smoothing windows, respectively. SP-WVD provides good cross-term suppression with independent adjustment of time and frequency resolutions [24,41]. The results of the SP-WVD did not gain much appreciation in the overall imaging results, as it still provides a significant amount of cross-term interference. A new technique (adaptive decomposition filter bank with SP-WVD) is proposed in this work in order to obtain more accurate micro-Doppler signature/pattern imaging. The proposed new approach extracts features (single-tone frequency and micro-Doppler bandwidth) from the power spectrum plot/statistics. Based on the values of these features, the filter bank parameters are instantaneously tuned (adapted) to the required frequency and bandwidth for which further decomposition must be performed. The proposed technique, i.e., the SP-WVD with an adaptive filter bank, yields a significant amount of cross-term interference attenuation in the overall micro-Doppler signature/pattern images. The pseudo-algorithm for the implementation of the proposed SP-WVD with the adaptive decomposition filter bank technique is given in the next section.

## 3. Design for RF Sensor and Experiments

**Figure 2:**
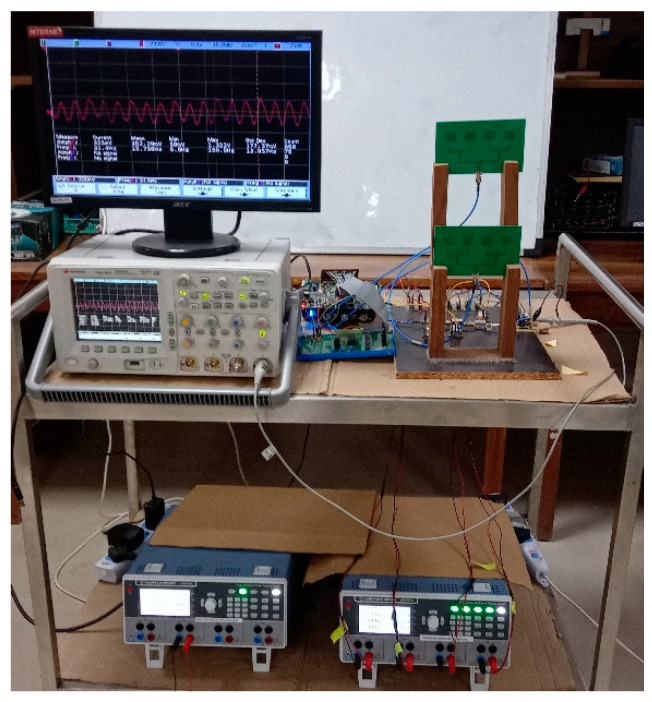
Photograph of the developed radio-frequency (RF) sensor’s experimental setup.

**Figure 3:**
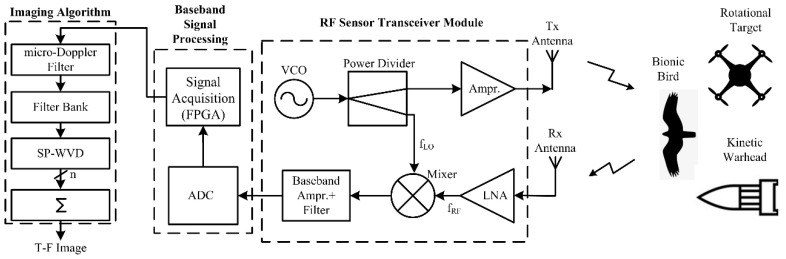
A schematic diagram illustrating the developed RF sensor.

A C-band CW RF sensor working at 5.3 GHz was built at our radar system design laboratory. The photograph of the designed RF sensor experimental setup is shown in Figure 2, and it consists of RF transmission (Tx)/reception (Rx) antennas, a transceiver module, baseband signal processing circuitry, and a micro-Doppler signature/pattern imaging algorithm. Two separate rectangular microstrip patch antennas (1 × 4 array) are used for RF signal transmission and reception. In the RF sensor’s transceiver module, the Tx chain consists of a voltage-controlled oscillator, a power divider, and a power amplifier for which its output is connected to the Tx antenna. The Rx chain obtains the raw echo signal from the Rx antenna and passes it through the low-noise amplifier, mixer, baseband amplifier, and low-pass filter (LPF) to filter out the high-frequency (f_LO_ + f_RF_) component [20], as shown in Figure 3. More details on the specifications of RF components/subsystems can be found in our recent publication [29]. The sensor is operated at a maximum power of 20 dBm. The output of the RF sensor transceiver module, i.e., high-frequency-filtered baseband sensor echo signal, is passed into an analog-to-digital (A/D) converter, which is driven by a master digital circuit implemented in a Xilinx field-programmable gate array (FPGA) where preliminary baseband signal processing [35,44] is performed.

**Figure 4:**
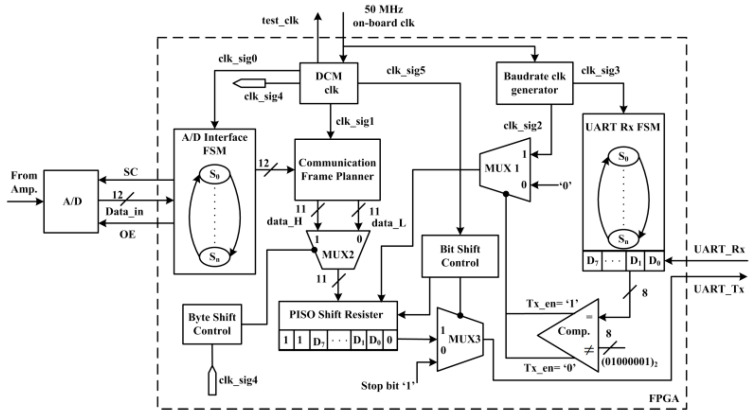
RF signal acquisition and preprocessing digital architecture built in the field-programable gate array (FPGA).

The pipelined digital architecture built in the FPGA for preprocessing [35,45] and the universal asynchronous receiver transmitter (UART) data transfer interface [35] are shown in Figure 4. The external A/D converter is interfaced to the FPGA via the start of conversion (SC), output-enable (OE), and data_in [11:0] ports. A digital clock manager (DCM) is designed inside the FPGA to have overall control on data conversion, data reading, data framing, and data transfer actions. The developed digital architecture consists of three different sections: A/D converter interface finite state machine (FSM), sensor data transfer UART_Tx engine, and synch-byte detector UART_Rx FSM. The detailed internal operations of this digital architecture can be found in our previous publications [19,35,45].

The preprocessed data are transferred into the computer at a baud rate of 115,200. In the computer, MATLAB collects the data samples in real-time, plots the time-domain signal, and generates a power spectrum and micro-Doppler signature/pattern images, as discussed in Section 2, for different outdoor experimental trials. The software algorithms collect the received signal for a pre-decided period, process it, update the time-/frequency-domain plots, and generate micro-Doppler signature/pattern images. The micro-Doppler resolution in this work is 0.5 Hz.

The pseudo-flows of computations associated with the software implementation of the proposed SP-WVD with an adaptive filter bank algorithm are as follows:

(a) Acquisition of RF sensor signal/data samples at 10 kHz: raw non-stationary multifrequency data/samples having the targets’ behavioral micro-Doppler signature/pattern profiles;
(b) Plotting the time series data of the RF sensor;
(c) Filtering the upper band frequency (>1 KHz) based on the radar’s Doppler bandwidth. A suitable LPF is designed as follows [35,43];
  (13) $$H_{LPF}(\omega )=\{\begin{array}{c} 1\;\mathrm{if}\;\omega \;\leq \omega_{c}\\ 0\;\mathrm{if}\;\omega_{c}<\omega \end{array}$$
(d) Computation and plotting of the power spectrum of sensor data using Equation (1);
(e) Extracting the main features from the power spectrum of sensor data (from the results of step (d) to decide/design the adaptive decomposition filter bank structure;
(f) Decomposition of signals. The response of the LPF is divided into two bands: 0–0.5 KHz using an LPF designed based on Equation (13) and 0.5–1 KHz using a bandpass filter (BPF) designed as follows [35,43];
  (14) $$H_{BPF}(\omega )=\{\begin{array}{c} 1\;\mathrm{if}\;\omega_{l}\leq \omega \;\leq \omega_{h}\\ 0\;\;\;\;\;\;\;\;\;\;\;\mathrm{otherwise} \end{array}$$
(g) Sub-level decomposition of the sensor signal: the required levels, within the Doppler band, are obtained using enough LPFs and BPFs, i.e., the decomposition filter bank structure;
(h) Responses of all LPFs and BPFs are linearly amplified using a constant gain of 20 dB;
(i) Amplified signals are passed through the SP-WVD algorithm (Equation (12)), and all computations are performed, in parallel, using parloop in the MATLAB environment;
(j) Summing the results of all SP-WVD channels: sum = { SP-WVD (LPF_1_) + SP-WVD (LPF_2_) + …..+ SP-WVD (LPF_x_) } + { SP-WVD(BPF_1_)+ SP-WVD(BPF_2_) +…….+ SP-WVD (BPF_y_) }, where x∈1,2,….H; y∈1,2,…..I; the values of *H* and *I* are integers and depend on the Doppler bandwidth;
(k) Micro-Doppler signature/pattern imaging based on the results of the summed SP-WVD.

**Algorithm 1.** Pseudo Code: Proposed SP-WVD with an adaptive filter bank
  1:
**begin**
  2:Input: 1D vector-RF sensor signal/data samples @ 10 KHz  3:Filtered: micro-Doppler filter: lowpass (Input)@ $\omega_{c}$ = 1 KHzIf $\omega \;\leq \omega_{c}$, $H_{LPF}$$(\omega )=1,\;\mathrm{else},\;H_{LPF}$(*ω*) = 0, end  4:Data: Base band Doppler signal   5:Spectrum: FFT (Data) @ nFFT: 65536  6:Parameter: Spectral features estimation and filter bank generation  7:Filters: lowpass (Parameter): step 2 & bandpass (Parameter):If $\omega_{l}\leq \omega \;\leq \omega_{h}$$,\;H_{BPF}$$(\omega )=1,\;\mathrm{else},\;H_{BPF}$(*ω*) = 0, end  8:Decomposition: Spectral decomposition: Filtering data  9:Amplification: Gain* Decomposition results  10:T-F Imaging: SP-WVD (Amplification)  11:Output: Sum (T-F Images)  12:Imaging: Plot (Output)  13:
**end**



The proposed Algorithm 1 is implemented in a classical computer with Windows 10, Intel Core i5-8250U CPU, 1.60GHz, 1.80GHz, and installed memory 12 GB. The computation complexity of the proposed technique is calculated and compared with the counterpart techniques in terms of processing time and physical memory (space). For STFT, CWT, SP-WVD, and proposed technique, processing time and physical memory (space) required are (1.32 s and 0.01 GB), (2.15 s and 0.051 GB), (6.0 sec and 0.55 GB), and (8 s and 0.95 GB), respectively. 

## 4. Results and Data Analysis

The experimental analysis of the combined (multifrequency) micro-Doppler effects of the targets in the developed RF sensor and signal processing algorithms is presented in this section. The targets, shown in Figure 1, were operated at different ranges in front of the sensor, corresponding experimental data were collected, and the results were verified using respective signature/patterns images. The experimental results and findings associated with all these investigations are reported below.

### 4.1. Experimental Micro-Doppler Signature/Pattern Generation Accuracy Comparative Analysis

**Figure 5:**
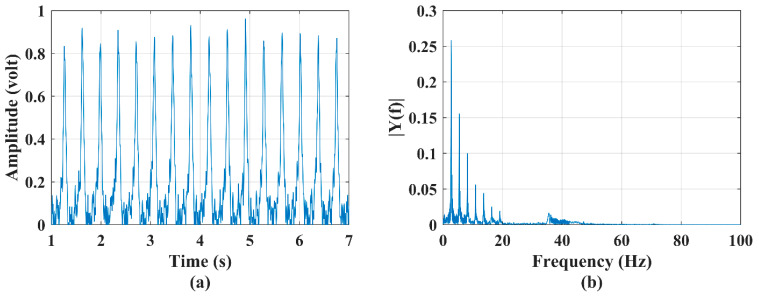
(**a**) Time−series plot and (**b**) power spectrum plot of a bionic bird’s sustained flapping action and a three−blade propeller system rotational motion.

**Figure 6:**
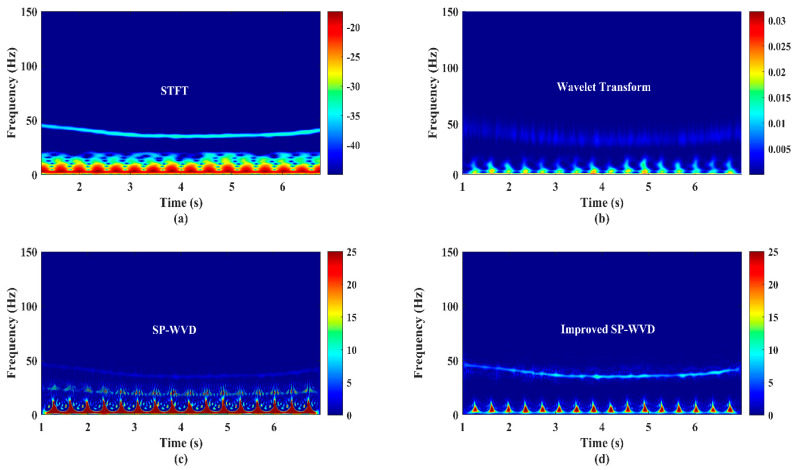
The bionic bird’s sustained−flight action’s and a three−blade rotational propeller system’s micro−Doppler signature/pattern image designed using (**a**) short−term Fourier transform (STFT), (**b**) wavelet transform, (**c**) SP−WVD and, and (**d**) improved SP−WVD.

In order to experimentally investigate the accuracy of the generation of micro-Doppler signature/pattern images using four different algorithms (STFT, CWT, SP-WVD, and proposed improved SP-WVD) considered in this work, a bionic bird (shown in Figure 1a) was operated (sustained flight at a point) in front of the RF sensor at a range of 40 m and at an altitude of 10 m. This scenario imitates one of the unique postures: the bionic bird’s at-a-point-sustained flight (unlike natural birds). Sensor data were collected in real-time, time-domain data are shown in Figure 5a, and the power spectrum computed using Equation (1) on the time-domain signal is shown in Figure 5b. The data corresponding to this experiment were applied to STFT, CWT, SP-WVD, and improved SP-WVD algorithms. The micro-Doppler signature/pattern images generated using the results of these three algorithms (Equations (3)–(6), Equations (7) and (8), and Equations (9)–(12)) and improved SP-WVD algorithms are shown in Figure 6a–d, respectively. In this experiment, the bionic bird was operated at a wing-flapping/wing-beating rate of 2.66 per second, as explained in Section 1, before the RF sensor.

Hence, the signature/pattern image shows 16 frequency spikes of 6 s duration in all signature/pattern images. The micro-Doppler spectrum is ~17 Hz, which depends on the length and flapping speed of the wings of the bionic bird [4,8,29]. The appearance of these 16 spikes in all the generated images evidences the detection accuracy of the developed RF sensor for the measurement of the wing-flapping/wing-beating rate of the bionic bird. However, in the aspect of the generation of accurate micro-Doppler signature/pattern images, the results of every algorithm differ in certain features/aspects. Due to the trade-off between window selection for time and frequency resolution in the STFT algorithm and the results shown in Figure 6a, (i) the time resolution of frequency spikes is not clear, (ii) frequency spectral distributions are not precise, and (iii) there is an inaccurate decomposition of multifrequency components over all time.

In the results of the CWT, shown in Figure 6b, almost all these constraints are observed, except for a slight improvement in the time resolution of frequency spikes, due to the limitations in the selection of the window length of the mother wavelet, the translation parameter value, and various levels of the scaling parameter for a specified range of dilations/contractions of the mother wavelet. In the results of the SP-WVD, shown in Figure 6c, all constraints are observed with reasonable accuracy, but there is strong cross-term interference. The results shown in Figure 6d attest to the precise detection and accurate micro-Doppler signature/pattern imaging using the developed RF sensor and improved SP-WVD without cross-term interference. The manual or automated measurement (count) of the bionic bird’s wing-flapping rate using the results shown in Figure 6d is straightforward and accurate. Similar results were obtained for all outdoor experimental trials in several cases with different types of low RCS targets shown in Figure 1. Therefore, only the experimental results obtained using improved SP-WVD are presented in the subsequent parts of this section.

### 4.2. Recognition of the Micro-Doppler Signature/Pattern of Two Targets’ Motions: Rotational Propeller System and Flapping Bionic Bird

The formulation of a micro-Doppler signature/pattern image for the simultaneous activities of two targets in front of the developed RF sensor is reported in this section. In this experiment, two different low RCS targets, a three-blade rotational propeller system, and a flapping bionic bird, shown in Figure 1a,b, respectively, were operated at a range of 50 m and 45 m, respectively. This experimental scenario imitates flying a three-blade propeller system drone and a bird in front of the sensor system. The RPM of the three-blade rotational propeller system and flapping speed of the bionic bird are varied by varying the control voltage via the given knobs, as discussed in Section 1. The values kept for these targets in this experiment are 1866 RPM and three flaps per second during 1–3 s and 4–7 s and two flaps per second during 3–4 s, respectively. 

**Figure 7:**
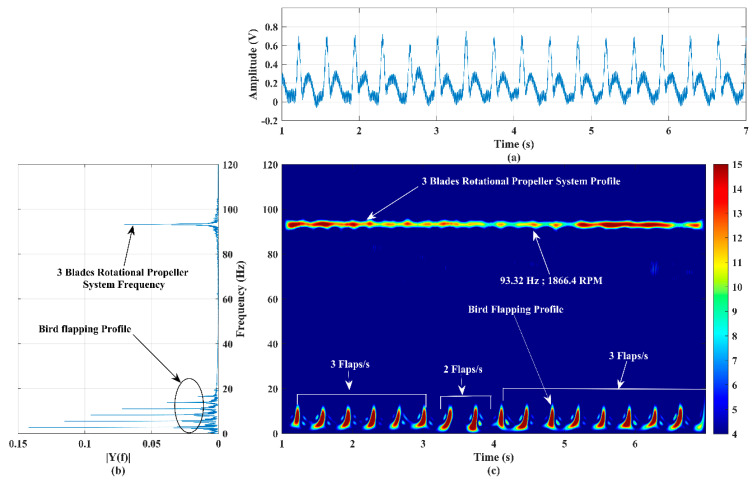
(**a**) Time−series plot, (**b**) power spectrum plot, and (**c**) micro−Doppler signature/pattern image designed using the improved SP−WVD for simultaneous activities of two targets: a three−blade rotational propeller system and a bionic bird’s flapping actions.

The time-domain signal and its power spectrum corresponding to this experiment are shown in Figure 7a,b, respectively. The power spectrum plot shows a band of frequency corresponding to the bird’s flapping action and a single tone corresponding to the rotation of the three-blade propeller system. The measured low-frequency band is ~17 Hz (DC-17 Hz), and the single-tone high frequency is 93.32 Hz. The time-domain data, shown in Figure 7a, were applied to the SP-WVD algorithm, and the results are shown Figure 7c, which clearly exhibits the activities of both targets over all measurement periods. The micro-Doppler signature/pattern image, shown in Figure 7c, shows a good correlation with the power spectrum shown in Figure 7b. Since the RPM of the rotational propeller system is maintained at a specific value (tachometer measured value is 1866 RPM), as detailed in Section 1, the micro-Doppler signature/pattern (93.32 Hz) observed in Figure 7c is unchanged throughout the experimentation period. The RPM of the propeller system is also computed using the micro-Doppler signature/pattern single-tone frequency (93.32 Hz), as discussed in [5], and the obtained value is 1866.4 RPM, which is in good agreement with the tachometer-measured value. The designed micro-Doppler signature/pattern image has good accuracy and clearly illustrates the flapping profile: three spikes during 1–3 s and 4–7 s and two spikes during 3–4 s. Thus, the results of the improved SP-WVD highly support the identification of combined activities of two different targets, precise computation of the RPM of the rotational propeller, and the detection of the bird’s flapping profile/speed.

### 4.3. Extraction of Micro-Doppler Signature/Pattern Profiles of Three Targets’ Motions: Three- and Two-Blade Rotational Propeller Systems and a Flapping Bird

To examine the micro-Doppler signature/pattern imaging accuracy of the developed RF sensor and the improved SP-WVD with three different (rotational and flapping) targets, a three-blade and a two-blade rotational propeller system and a bionic bird were operated in front of the sensor at a range of 15 m, 20 m, and 30 m, respectively. 

**Figure 8:**
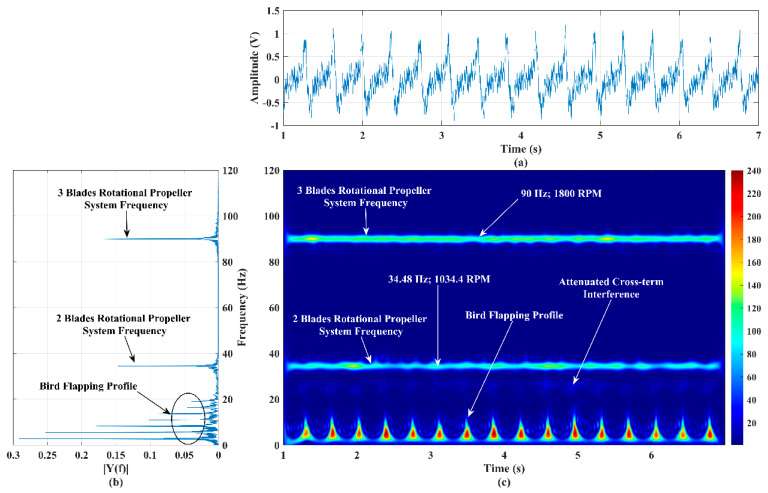
(**a**) Time−series plot, (**b**) power spectrum plot, and (**c**) micro−Doppler signature/pattern image designed using the improved SP−WVD for the simultaneous activities of three targets: two−blade and three−blade rotational propeller systems and a bionic bird’s flapping actions.

This scenario imitates the simultaneous activities of multiple drone-like targets (having a rotational portion) and a bird’s movement in front of the RF sensor. In this experiment, the two-blade and three-blade rotational propeller systems were operated at the tachometer-measured RPM of 1034 and 1800, as discussed in Section 1, respectively. The flapping rate of the bionic bird was randomly varied for the purpose of studying the detection and imaging capability of the developed sensor and algorithm for these random variations. The time-domain signal and its power spectrum corresponding to these targets activities are shown in Figure 8a,b, respectively. The power spectrum shows a low-frequency band (~DC-18 Hz) for the bird’s flapping, a single-tone frequency of 34.48 Hz for the two-blade propeller system’s rotations, and 90 Hz for the three-blade propeller system’s rotations. The time-domain signal shown in Figure 8a is applied to the improved SP-WVD. The generated micro-Doppler signature/pattern is shown in Figure 8c, which correlates with the power spectrum shown in Figure 8b.

The RPM of both propellers’ rotations are also computed using the respective micro-Doppler frequency perceived in Figure 8c (34.48 Hz (for two blades) and 90 Hz (for three blades)), and the corresponding RPM values are 1034.4 and 1800, respectively. The developed RF sensor-based measurement shows good agreement with the tachometer’s measurements. The bird’s random flapping speed is clearly reflected/noticed in Figure 8c, which evidences the capability of the developed RF sensor for the accurate detection of a bionic bird operating/flying at a random flapping speed. Furthermore, the results of this experiment, shown in Figure 8b,c, show that the attenuated cross-term interference falls well below the strength of micro-Doppler signatures, which supports the accurate detection of activities of multiple low RCS targets. However, optimizing the algorithm, aiming to completely remove the cross-term interference, is ongoing research at our RF sensor laboratory.

### 4.4. Signature/Pattern Imaging of a Static Rotational (Propeller System) and a Guided Orbital Motion Target

**Figure 9:**
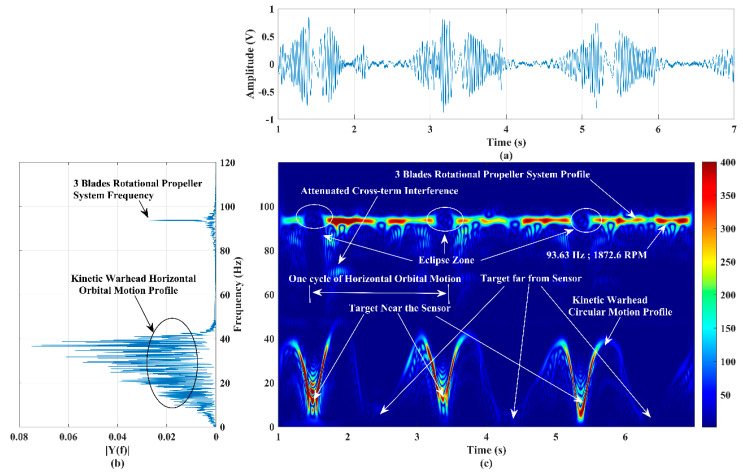
(**a**) Time−series plot, (**b**) power spectrum plot, and (**c**) micro−Doppler signature/pattern image designed using the improved SP−WVD for the simultaneous activities of two targets: a three−blade rotational propeller system and a circular horizontal path kinetic warhead’s orbital motion.

The detection accuracy of the developed RF sensor system was examined by operating two different targets: a three-blade propeller system rotating at a range of 20 m and a horizontal-guided (on the plane of the sensor boresight/beam) kinetic warhead (shown in Figure 1d) orbiting about the range of 15 m. This scenario imitates the simultaneous operation of a drone target (having a rotational portion) for guiding the surveillance (in a circular path) kinetic warhead (having no rotational portion) target. The tachometer-measured the RPM of the three-blade propeller system; in this case, it is 1873, and the kinetic warhead is randomly (angular velocity and radius of the circular path are not constant) operated about the range of 15 m. The time domain and power spectra of this experiment are shown in Figure 9a,b, respectively. The power spectrum clearly illustrates a frequency band (DC-42 Hz) and a single-tone frequency (93.63 Hz) corresponding to the kinetic warhead’s circular orbital motion and rotation of the three-blade propeller system, respectively.

The micro-Doppler signature/pattern image generated using the RF system is shown in Figure 9c, and it clearly evidences the behavioral profile of these two targets. As discussed in [29], the computed RPM using 93.63 Hz is 1873, which is the same as the tachometer’s value. The low-frequency micro-Doppler signature/pattern shown in Figure 9c explores the orbital circular random motion of the kinetic warhead target. Each cycle’s half-frequency profile corresponds to half of the circular path orbital motion. The sensor echo power increases as the target approaches the sensor. The signature/pattern image absolutely follows the orbital circular path motion of the kinetic warhead target.

**Figure 10:**
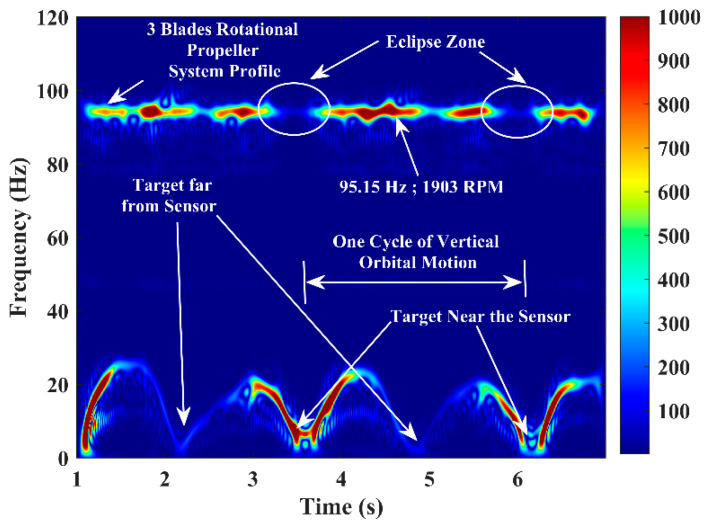
Micro−Doppler signature/pattern image designed using the improved SP−WVD for the simultaneous activities of two targets: a three−blade rotational propeller system and a circular vertical path kinetic warhead’s orbital motion.

The same experiment was conducted with a vertical path (sensor beam/boresight plane) orbital motion kinetic warhead target, and the corresponding micro-Doppler signature/pattern image is shown in Figure 10. In this trial, the RPM of the three-blade propeller system is 1903, and the kinetic warhead operation is random. The experimental trials were conducted with different aspect-angle-tilted (with an angular tilt to the sensor beam/boresight) circular/elliptical/figure-of-eight orbital paths and corresponding micro-Doppler signature/pattern images. 

These signatures clearly confirm the simultaneous behavioral detection of low RCS aerial targets having rotational and non-rotational parts. However, when the kinetic warhead appears close to the radar in the boresight axis, it completely blocks the echo power of the rotational propeller system target due to the target’s size difference. 

This phenomenon introduces an eclipse in all experimental trials, as in Figure 9c and Figure 10, and this is challenging since a small-size aerial target can be operated, hiding it behind a slightly larger-sized aerial target. The eclipse can be avoided, and such hidden targets can be detected by establishing the RF sensor network to look at the region of interest from different angles/points.

### 4.5. Behavioral Imaging of Activities of Three Different Targets: Three-Blade Propeller Rotation System, Kinetic Warhead Motion, and Bird’s Flapping Action

**Figure 11:**
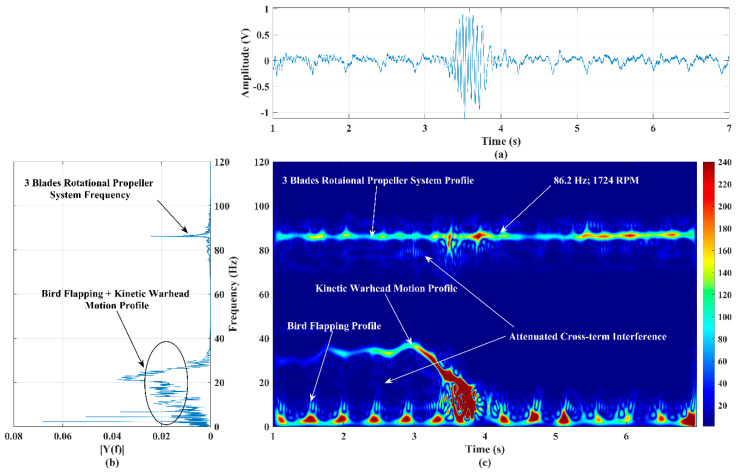
(**a**) Time−series plot, (**b**) power spectrum plot, and (**c**) micro−Doppler signature/pattern image designed using improved SP−WVD algorithm for the simultaneous activities of three targets: flapping bird, three−blade rotational propeller system, and kinetic warhead linear path motion.

In this experimental trial, three different targets (bionic bird flapping, three-blade propeller system rotation, and kinetic warhead structure motion) were simultaneously performed in front of the sensor at ranges of 30 m, 37 m, and 45 m, respectively. During this experiment, sustained rotational (tachometer value is 1724) and flapping (random) actions were performed, and the kinetic warhead was moved from 45 m to 42 m at a velocity of ~1.09 m/s. The translational motional speed of this target decreases at a range of 43 m as it approaches the sensor. The time-domain signal and power spectrum results corresponding to this experimental trial are shown in Figure 11a,b, respectively. As long as the apex of the kinetic warhead structure target is in the sensor’s boresight plane/axis, the amplitude profile of the time-domain signal is almost uniform (within ±2 V) during the first 2 s. A significant amount of amplitude fluctuations, as shown in Figure 11a, is observed during 3–4 s, when the target exposes its curved surface to the sensor while deviating its motional path to come out of the sensor’s beam. The generated micro-Doppler signature/pattern image for this experiment is shown in Figure 11c. The sensor echo multifrequency signal decomposition accuracy of the improved SP-WVD can be clearly observed in Figure 11b,c. 

The micro-Doppler signature/pattern image distinguishably illustrates the frequency profiles of the activities of all these targets: rotation of a propeller system, flapping of a bird, and linear path motion and exit of a kinetic warhead structure. The results evidently explore the effect of the kinetic warhead while it exits the RF sensor beam. The computed RPM using the rotational micro-Doppler frequency (86.2 Hz) is equal to a tachometer value of 1724 RPM. The motional Doppler maximum frequency observed from Figure 11c is ~39 Hz, which is due to the linear path motion of the kinetic warhead at a velocity of ~1.09 m/s.

## 5. State-of-the-Art (SOTA) Comparative Discussion

To clarify the improved performance of the developed RF sensor and the proposed micro-Doppler signature/pattern imaging technique (algorithm), we report a comparison of relevant state-of-the-art (SOTA) micro-Doppler signature extraction microwave radar sensors and imaging techniques found in the literature with the results of our sensor/technique in this section. As we mentioned in Section 1, no literature discussing the combined micro-Doppler effects of multiple simultaneously operated low RCS flapping/rotational/warhead-structure-motional targets is found; hence, the experimental results presented in this paper are unique. Therefore, performing a one-to-one mapped SOTA analysis becomes impossible. However, the capability and accuracy of the developed RF sensor and the proposed imaging technique confirmed via a series of open-field trial experiments with different low RCS targets are compared with relevant SOTA literature. The SOTA comparison results are given in Table 2, which covers the literature on the extraction/imaging of the microwave-sensor-based micro-Doppler signature of a flapping bird, a rotational propeller system, and warhead structure targets. 

Commercial CW or pulsed RF sensor ready-made modules (relatively high cost), at the available frequency, are used in most of the relevant literature. Furthermore, pulsed radar or FMCW radar is used wherever the range is a matter of interest in the study. In the literature, a more common as well as simple STFT technique is used to generate micro-Doppler signature/pattern images, except in [51,53], where the direct SP-WVD- and STFT-based scattering center reconstruction method, respectively, are used. Several outdoor experiments were conducted to examine the performance of the developed RF sensor and the proposed adaptive-filter -bank-assisted SP-WVD algorithm, and we obtained appreciable, accurate results, as expected, in all trials compared to relevant SOTA results.

## 6. Conclusions

This paper describes the significance of detecting and imaging targets’ micro-Doppler signatures/patterns. The designs of an RF sensor (C-band 5.3 GHz) and a digital circuit built inside the FPGA are described. An innovative approach is proposed with the SP-WVD and adaptive decomposition filter bank, and its results are validated against three standard techniques: STFT, CWT, and SP-WVD. Different types of targets (two-/three-blade propeller systems, bionic bird, and kinetic warhead) exhibiting rotational/flapping/kinetic warhead motional profiles/signatures are remotely controlled/operated in front of the sensor. Applicability of the developed RF sensor with the proposed innovative approach (SP-WVD with an adaptive decomposition filter bank) for generating accurate micro-Doppler signature/pattern images is demonstrated in different open-field trials. The RPM measurements and generated micro-Doppler signature/pattern images are always close to 100% accuracy against the measurement values and activities of the targets, respectively.

The computational complexity of the proposed technique can be reduced when implementing it in the CUDA processor/core, higher-end workstation, or in digital platform like FPGA, which is one of our ongoing research works. Furthermore, near-future work should address (i) finding a more suitable hybrid algorithm to completely attenuate cross-term interference, particularly when the low RCS aerial targets form a cluster (wider group); (ii) extracting range information by modulating the CW sensor; (iii) designing an RF sensor to accommodate a wide spectrum of micro-Doppler bandwidths (high-speed angular/radial frequency) with an increased range/Doppler frequency resolution; (iv) determining the length/width of the propeller blades, bird’s wings, and kinetic warhead; (v) establishing a drone detection sensor network to look at the area of interest from multiple angles to detect the activities of drone-hidden targets; and (vi) performing automated targets’ behaviors’ classification/recognition using artificial intelligence (AI)/machine learning (ML) techniques, which are not applied in this sensor system. 

## Figures and Tables

**Table 1:** Specifications of the low RCS flapping/rotational/motional targets used in this work.

S. No.	Target	Specification
1	Bionic bird (Figure 1a)	Flapping speed: 1–4 flap/s; wing length: 19 cm; bird’s body width: 5.5 cm; wing beat control: 3 V DC motor; flap control voltage: 1.9–3.0 V; control provision: pot. knob variation; RCS [42]: ~−28.86 dBsm
2	3-Blade propeller (Figure 1b)	Blade length: 7.5 cm; maximum blade width: 7 cm; blade thickness: 0.1 cm; maximum revolutions per minute (RPM): 1910; blade propulsion: 5 V DC motor; RPM control provision: pot. knob variation; RPM control voltage: 0.5–5 V; RPM measurement: using a tachometer; sensor frequency to RPM conversion: RPM = (fx60)/n [25,29]; RCS [42]: ~−0.268 dBsm.
3	2-Blade propeller (Figure 1c)	Blade length: 20 cm; maximum blade width: 4.5 cm; blade thickness: 0.1 cm; maximum RPM: 1740; blade propulsion: 230 V AC motor; RPM control provision: silicon control rectifier (SCR) knob variation; RPM control voltage: 100–230 V; RPM measurement: using a tachometer; sensor frequency to RPM conversion: RPM = (fx60)/n [25,29]; RCS [42]: ~9.77 dBsm
4	Kinetic warhead (Figure 1d)	Height: 16 cm; base radius: 7 cm; mass: 1 kg; mounting: screw with fuel propeller system; nose alignment: 3-axis tip tilt; RCS [42]: ~−41.87 dBsm.

**Table 2:** State-of-the-art (SOTA) comparison of flapping, propeller rotation, and warhead-structure-motional micro-Doppler signature/pattern extraction/imaging sensors/techniques.

S. No.	Type of Target	Authors	RF Sensor	Imaging Technique	Improvements Attended Using the Proposed Sensor/Technique	Comparative Remark
1	Flapping bird	Molchanov, P., et al. [4]	Continuous-wave (CW) sensor (9.5 GHz)	STFT	Maximum micro-Doppler: 1 kHz Maximum flapping speed: 28 m/s Frequency resolution: ~0.5 Hz Time resolution: ~0.08 s Sensor: CW (5.3 GHz) Maximum range: 100 m Target horizontal orientation: 0°–360° Target vertical orientation: 0°–360° Target behaviors: sustained flapping/flying/gliding	Improved performance is obtained using the proposed RF sensor and technique than the SOTA results.
2		Rahman, S., et al. [5]	CW sensor (24 GHz and 94 GHz)			
3		Farshchian, M., et al. [6]	CW sensor (24 GHz)			
4		Chen, V.C. [46]	Frequency-modulated continuous-wave (FMCW) sensor (X-band)			
1	Rotational propeller system	Rahman, S., et al. [5]	CW sensor (24 GHz and 94 GHz)	STFT	Maximum micro-Doppler: 1 kHz Maximum rotational frequency: 1 kHz Maximum RPM: 20,000 (for 3 blades), 30,000 (for 2 blades) Frequency resolution: ~0.5 Hz Sensor: CW (5.3 GHz) Maximum range: 100 m Target vertical orientation: −90°–+90° Target behaviors: sustained flying/tilted flying	Improved performance is obtained using the proposed RF sensor and technique than the SOTA results.
2		Vishwakarma S., et al. [47]	CW sensor (7.5 GHz)			
3		Fioranelli, F., et al. [48]	Pulsed sensor (2.4 GHz)			
4		Kim, B.K., et al. [49]	FMCW sensor (14.03–14.18 GHz)			
1	Motorized laboratory model kinetic warhead structure	Choi, I., et al. [50]	Pulsed sensor (10 GHz; PRF = 1 kHz)	STFT	Maximum micro-Doppler: 1 kHz Maximum angular velocity: 6.28 E3 rad/s Frequency resolution: ~0.5 Hz Sensor: CW (5.3 GHz) Maximum range: 100 m Target horizontal orientation: −45° to +45° Target vertical orientation: −45° to +45° Eclipse sensing: yes Tilted orbital path: Yes −30° to +30° Orbital path: circular or arbitrary Target behaviors: horizontal/vertical orbital motion, projectile motion, spinning	Improved performance is obtained than the SOTA results’ expected range. Since [47,48] show simulations, the target range is in the order of kilometers.
2	Simulation model of a ballistic missile warhead-structure-spinning profile	Sun, H., et al. [51]	Pulsed sensor (10 GHz; PRF = 250 Hz)	SP-WVD		
3	Simulation model of a warhead-structure-spinning profile	Jung, J., et al. [52]	Pulsed sensor (10 GHz; PRF = 6 kHz)	STFT		
4	Coning target model warhead structure	He, F., et al. [53]	Pulsed sensor (10 GHz; PRF = 5 Hz)	STFT-based scattering center reconstruction method		

## Data Availability

The data presented in this study are available upon request from the corresponding author. The data are not publicly available due to relevant ongoing research.
